# Dietary Brown Mushroom Stem Powder Modulates Ileal Morphology and Barrier-Related Gene Expression in Layer Chicks

**DOI:** 10.3390/ani16142254

**Published:** 2026-07-21

**Authors:** Md Salahuddin, Prantic Kumar Goswami, Ahmed A. A. Abdel-Wareth, Kayla G. Stamps, Andrés Pech-Cervantes, Mustafa Hitit, Jayant Lohakare

**Affiliations:** 1 Poultry Center, College of Agriculture, Food and Natural Resources, Prairie View A&M University, Prairie View, TX 77446, USA; mdsalahuddin@pvamu.edu (M.S.); pgoswami@pvamu.edu (P.K.G.); aahabdelwareth@pvamu.edu (A.A.A.A.-W.); kstamps2@pvamu.edu (K.G.S.); 2 International Goat Research Center, Cooperative Agricultural Research Center, Prairie View A&M University, Prairie View, TX 77446, USA; aapechcervantes@pvamu.edu (A.P.-C.); muhitit@pvamu.edu (M.H.)

**Keywords:** brown mushroom stem, gut barrier, ileum morphology, tight junction genes, layer chicks

## Abstract

Brown mushroom stem (BMS), a nutrient-rich by-product of the mushroom processing industry, represents a potential sustainable feed ingredient for poultry. However, its effects on intestinal morphology and barrier-related gene expression in layer chicks are not well characterized. This study evaluated the influence of dietary BMS on ileal morphology and intestinal barrier-related gene expression in layer chicks. A total of 160 Lohmann LSL Lite chicks were randomly allocated to 4 dietary treatments: a control diet and control diets containing 2%, 4%, or 6% BMS as partial replacements for soybean meal on an equivalent basis, respectively. Each treatment had 5 replicates with 8 birds per replicate, and the trial lasted 36 d. Ileal samples were collected for histomorphological evaluation and quantitative real-time PCR of barrier-associated genes, including claudin-1 (*CLDN1*), occludin (*OCLN*), tight junction protein 1 (*TJP1*), tight junction protein 2 (*TJP2*), and mucin-2 (*MUC2*). Data were analyzed using one-way ANOVA, and orthogonal polynomial contrasts were applied to determine linear and quadratic responses to increasing BMS inclusion levels. Villus height decreased in BMS-fed groups compared with the control (linear and quadratic effects, *p* < 0.001), whereas crypt depth showed a quadratic response with the lowest value at 4% BMS (*p* < 0.001). Expression of *CLDN1* mRNA was significantly elevated at 2% BMS but declined at higher inclusion levels (quadratic effect, *p* = 0.001). In contrast, *TJP2* mRNA expression decreased with increasing BMS inclusion (linear effect, *p* = 0.009). No significant differences were observed for *OCLN*, *TJP1*, or *MUC2* mRNA expression (*p* > 0.05). A composite index of barrier-related gene expression was numerically the highest at 2% BMS supplementation but did not differ significantly among treatments. These findings suggest that low dietary inclusion of BMS may support intestinal barrier-related gene expression in layer chicks, although higher levels may alter intestinal morphology without corresponding changes in barrier-related gene expression. Optimizing BMS inclusion could support sustainable poultry production by utilizing mushroom processing by-products as an alternative feed ingredient.

## Simple Summary

Mushroom processing creates large amounts of stems that are often discarded, but these stems still contain useful nutrients. We tested whether adding brown mushroom stems to the diet of young chicks could support gut health while reducing the need for soybean meal, a common but costly feed ingredient. One hundred sixty-layer chicks were fed diets containing either no mushroom stems or a low, medium, or high level of mushroom stems for 36 days. At the end of the study, we examined the structure of the small intestine and measured genes related to the gut barrier, which help prevent harmful microbes and toxins from crossing into the body. Chicks fed mushroom stems had shorter intestinal villi, which are finger-like structures that absorb nutrients. The depth of the intestinal crypts, where new gut cells are produced, was the lowest at the medium level of mushroom stems. The key barrier gene increased at the low mushroom stem level but dropped at higher levels, while another barrier gene decreased as mushroom stem levels increased. Other barrier genes did not change. Overall, a low level of mushroom stems showed the most favorable gut barrier-related gene response. These results suggest mushroom stems can be used in small amounts to beneficially influence gut barrier-related genes and improve sustainability by turning a food-processing by-product into poultry feed.

## 1. Introduction

Soybean meal (SBM) remains the primary protein source in poultry diets; however, its price volatility, reliance on global trade, and land-use footprint have intensified the search for sustainable and circular alternatives [1,2,3]. Contemporary feed markets are increasingly encouraging partial soymeal replacement strategies to improve economic resilience and environmental sustainability while maintaining performance and gut integrity. Achieving this goal requires evaluating not only growth and feed efficiency but also intestinal morphology and barrier function when introducing novel co-products [4]. Consequently, attention has shifted toward agro-industrial residues with functional bioactivity, including mushroom-derived by-products such as stems and trimmings [5].

Mushroom residues are rich in structural polysaccharides, particularly β-glucans, along with polyphenols and terpenoids that exert antioxidant and immunomodulatory effects and influence gut microbial ecology [6,7]. These bioactive compounds have been shown to improve feed efficiency, immune responses, oxidative stability, pathogen resistance, and product quality in poultry when administered as whole mushrooms or extracts [8,9]. Their ability to modulate intestinal microbiota and inflammatory responses has also positioned mushrooms as functional feed ingredients with potential gut-health benefits [7]. However, mushroom stems differ from extracts in that they contain higher levels of structural fiber, which may alter intestinal architecture in addition to providing bioactive compounds. Therefore, evaluating both structural and molecular outcomes is essential when incorporating mushroom stem powder into poultry diets.

The avian gastrointestinal tract (GIT) plays a central role in growth and immune development, particularly during early life [10]. The small intestine, as the primary site of digestion and nutrient absorption, is highly responsive to dietary composition, and alterations in feed formulation can significantly modify its morphology and functional efficiency [11,12]. Differences in intestinal structure can directly influence nutrient assimilation and production performance even under standardized management conditions [13]. Villus height and crypt depth are widely used histological indicators of intestinal health. Villus height reflects absorptive surface area, whereas crypt depth indicates epithelial turnover and proliferative activity [14,15]. Deeper crypts often signal increased tissue renewal demands, as epithelial cells originate in the crypt and migrate upward along the villus before being shed into the lumen [15,16]. Thus, optimal intestinal morphology reflects a balance between absorptive capacity and controlled epithelial turnover.

Beyond structural parameters, intestinal barrier integrity is critical when evaluating novel feed ingredients. The intestine must simultaneously absorb nutrients and prevent the translocation of pathogens and toxins. Tight junctions (TJ) serve as critical elements of the epithelial barrier, anchoring to the actin cytoskeleton [17,18,19]. These dynamic structures regulate paracellular permeability and respond to luminal stimuli, nutrients, and inflammatory mediators [20]. Disruption of TJs is associated with increased intestinal permeability, immune activation, and compromised performance [21]. Therefore, assessing tight junction gene expression provides insight, at the transcriptional level, into how dietary inclusion may influence the expression of genes associated with epithelial barrier integrity.

Recent evidence suggests that soybean meal inclusion can be reduced when alternative ingredients possess favorable nutrient composition and functional gut-health properties [22]. However, despite growing interest in mushroom stem utilization, dose–response data evaluating both intestinal morphology and barrier-associated gene expression in layer-type chicks remain limited. Early investigations have demonstrated the feasibility of stem inclusion but have not resolved the optimal inclusion level that preserves epithelial architecture while enhancing or maintaining barrier signaling. In particular, the interaction between structural adaptation and tight junction modulation under graded mushroom stem inclusion remains poorly defined [7,23].

Therefore, the present study was conducted to evaluate the dose-dependent effects of brown mushroom stem (BMS) powder as a partial soybean meal replacer on ileal morphology and barrier-related gene expression in layer-type chicks. We hypothesized that moderate BMS inclusion would selectively modulate the expression of tight junction-related genes without inducing adverse structural changes. In contrast, excessive inclusion could promote architectural adjustment without corresponding changes in barrier-related gene expression. To test this hypothesis, we assessed villus height, crypt depth, goblet cell density, and the relative expression of key tight junction genes and *MUC2* in the ileum.

## 2. Materials and Methods

All experimental procedures were conducted at the Poultry Center, Prairie View A&M University (PVAMU), Prairie View, TX, USA. Bird handling and experimental methods complied with the Animal Welfare Act (AWA), USDA Animal Welfare Regulations (AWRs), and the Guide for the Care and Use of Laboratory Animals. All experimental procedures were approved by the Prairie View A&M University Institutional Animal Care and Use Committee (PVAMU IACUC) under Animal Use Protocol (AUP) #2023-055 (approved on 5 July 2024).

### 2.1. Preparation of Brown Mushroom Stem (BMS) Powder

Brown mushroom stems were obtained from a local commercial source (Monterey Mushrooms, Madisonville, TX, USA). The collected material was washed, freeze-dried (Harvest Right, North Salt Lake, UT, USA), and ground into a fine powder prior to incorporation into diets. The detailed preparation method and chemical composition have been previously reported [23].

### 2.2. Birds, Housing, Diets, and Experimental Design

The experimental design, bird management, and husbandry procedures followed the approach described in our previously published study [23]. Briefly, a total of 160 one-day-old Lohmann LSL Lite layer chicks (Hy-Line North America, Bryan, TX, USA) were brooded on a commercial starter diet for 16 d. Birds were then fed a control diet for an additional 4 days of adaptation period. The intervention was initiated at 3 weeks of age so that birds had progressed beyond the initial, rapid phase of post-hatch intestinal development, during which villus and crypt architecture and barrier maturation are driven primarily by intrinsic developmental programming; this provided a stable morphological and functional baseline against which dietary effects could be assessed. The standardized common starter and adaptation periods further ensured a uniform baseline gut status across all birds before treatments were imposed. At 3 weeks of age, chicks were randomly allocated to one of four dietary treatments for 36 d using a completely randomized design: Control (0% brown mushroom stem; BMS), T1 (2% soybean meal replacement with BMS), T2 (4% soybean meal replacement with BMS), and T3 (6% soybean meal replacement with BMS). The 36-day feeding duration (from 3 to 8 weeks of age) was selected to encompass multiple cycles of intestinal epithelial renewal, allowing adaptive changes in intestinal morphology and barrier-related gene expression to develop and stabilize, while also corresponding to the active rearing phase of layer-type pullets. BMS was used to replace soybean meal at the specified inclusion levels on a 100% equivalent basis. Each treatment consisted of 5 replicates with 8 chicks per replicate (n = 40 chicks/treatment; total n = 160).

Chicks were housed in an environmentally controlled brooder facility in wire battery cages (five-tier system; GQF Manufacturing, Savannah, GA, USA) with ad libitum access to feed and water. Lighting was provided continuously (24 h) throughout the experiment, and room temperature was managed according to breed guidelines. Experimental diets were formulated at the Texas A&M University Feed Mill (College Station, TX, USA) to be isocaloric and isonitrogenous and to meet or exceed nutrient requirements for layer chicks [23]. The ingredients and calculated chemical composition of the experimental diets are presented in Table A1. Diets were prepared and provided in crumbled form. Feed was provided weekly, and feed intake was determined by recording feed offered and subtracting residual feed remaining at the end of each week. Intake was expressed on a replicate basis. Body weight gain, feed intake, and feed conversion ratio for these birds have been reported previously [23]; the present study focuses specifically on ileal morphology and barrier-related gene expression, and these growth performance data are therefore not reproduced here to avoid duplication of previously published results.

### 2.3. Sample Collection

Birds were not subjected to feed withdrawal prior to slaughter, and feed remained available *ad libitum* until the time of sample collection to preserve luminal content and to avoid fasting-induced changes in intestinal morphology and barrier-related gene expression. All samples were collected within the same time window of the day across treatment groups to minimize diurnal variation. At the end of the 36-day feeding period, two birds were randomly selected and sampled from each replicate cage (5 replicate cages per treatment; n = 10 birds per treatment) and humanely slaughtered for tissue collection. The ileum was carefully excised from each bird. The contents of the ileum were gently removed by flushing with 0.75% sodium chloride solution. For histomorphological evaluation, the distal ileum was immediately fixed in Bouin’s solution (Cat. No. 22-110-621, Fisher Scientific, Waltham, MA, USA) at room temperature for 24 h, following the protocol described by Salahuddin et al. [24]. For gene expression analysis, the proximal ileum was longitudinally opened, and the mucosal layer was carefully scraped using a sterile glass slide. The collected mucosal samples were immediately snap-frozen in liquid nitrogen and subsequently stored at −80 °C until RNA extraction and molecular analysis.

### 2.4. Morphometrical Analysis

**Figure 1:**
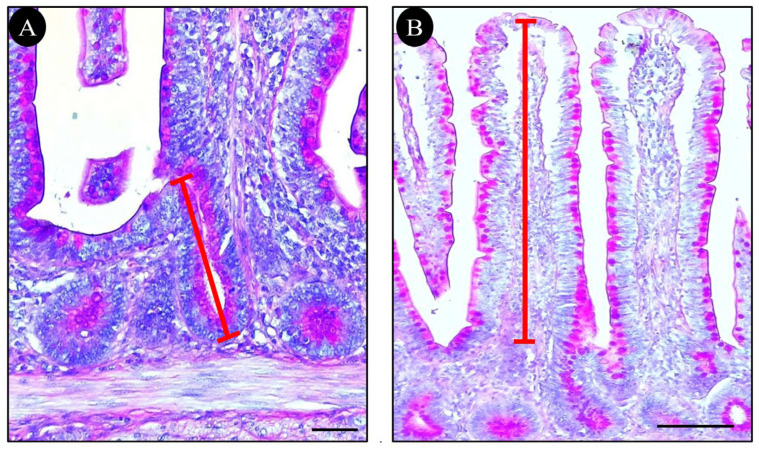
Representative photomicrographs of ileal histomorphology in layer chicks. (**A**) Measurement of crypt depth (CD), defined as the distance from the base of the crypt to the villus-crypt junction. (**B**) Measurement of villus height (VH), defined as the distance from the villus tip to the villus-crypt junction. Red lines indicate the regions used for morphometric measurements. Scale bars represent 100 µm in (**A**) and 50 µm in (**B**).

Fixed ileal tissues were processed using standard paraffin embedding procedures. Briefly, samples were dehydrated in graded ethanol, cleared in xylene, and embedded in paraffin wax. Sections were cut at 5 µm thickness using a rotary microtome (Leica RM 2135, Wetzlar, Germany) and mounted on glass slides. Sections were stained using the periodic acid-Schiff (PAS) reaction with Mayer’s hematoxylin (Cat. No. 22-050-111, Fisher Scientific, USA) counterstaining to allow visualization. Morphometric analysis was performed using a light microscope (Revolve R4, Discover Echo Inc., San Diego, CA, USA). Villus height (VH) was measured from the villus tip to the villus-crypt junction (Figure 1). Only well-oriented, intact villi were selected for measurement to ensure accuracy. Thirty villi were measured per bird, and a bird-level mean was calculated for each individual. Crypt depth (CD) was measured as the depth of the invagination between adjacent villi, extending from the base of the crypt to the villus-crypt junction (Figure 1), with 30 crypts measured per bird and a bird-level mean calculated accordingly. Measurements were performed consistently across all treatment groups, and replicate means were used as the experimental unit.

**Figure 2:**
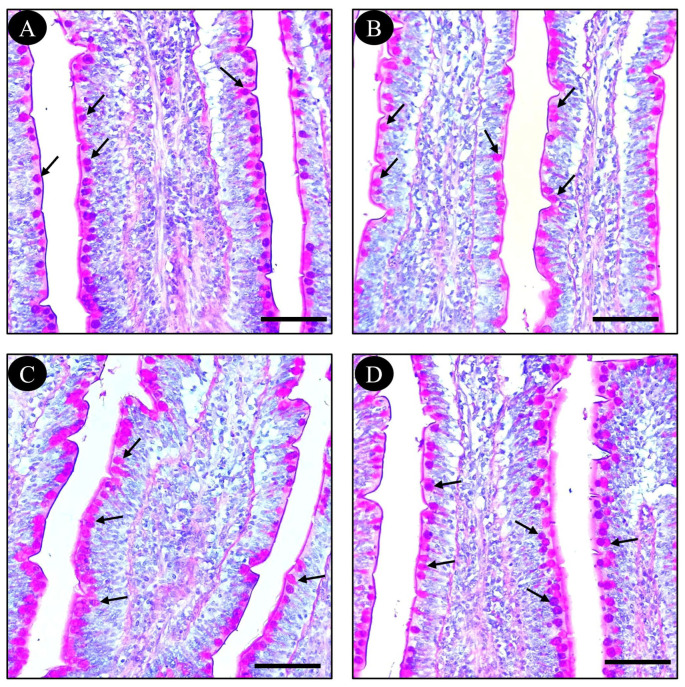
Representative PAS-stained sections of the ileum showing goblet cells in layer chicks fed diets containing different levels of brown mushroom stem (BMS). (**A**) Control (0% BMS), (**B**) 2% BMS, (**C**) 4% BMS, and (**D**) 6% BMS. Goblet cells are indicated by black arrows and appear as PAS-positive stained cells within the villus epithelium. Scale bars represent 50 µm.

Goblet cells were identified by their positive PAS staining and characteristic mucin-filled appearance within the villus epithelium (Black arrows, Figure 2). For each bird, thirty villus epithelial surfaces were randomly selected from photomicrographs. Goblet cells were counted along with each selected villus epithelium, and the corresponding epithelial surface length was measured using image analysis software (ImageJ, version 1.54t, National Institutes of Health, Bethesda, MD, USA). Goblet cell density was expressed as the number of goblet cells per 100 µm of villus epithelial surface length. A bird-level mean was calculated from the 30 villus surfaces measured per bird, and the mean of the two birds sampled per replicate cage was then used as the experimental unit, consistent with the villus height and crypt depth analyses. Thus, although a total of 300 villus surfaces were evaluated per treatment group, these were not treated as independent observations; the replicate cage served as the experimental unit, giving five experimental units per treatment.

### 2.5. RNA Extraction and Quantitative Real-Time PCR

Total RNA was extracted from ileal mucosal scrapings using TRIzol reagent (Cat. 15596026, Thermo Fisher Scientific, USA). Briefly, samples were homogenized using a bead beater (Bead Ruptor Elite^TM^, Omni International, Kennesaw, GA, USA) in TRIzol lysis reagent, followed by phase separation, RNA precipitation, washing, and resuspension according to standard TRIzol procedures. RNA concentration and purity were determined using a NanoDrop One^C^ spectrophotometer (Thermo Fisher Scientific, Waltham, MA, USA), and samples with acceptable purity ratios (A260/A280) were used for downstream analysis. RNA integrity was verified by 1% agarose gel electrophoresis, and only samples showing clear, intact 28S and 18S ribosomal RNA bands and no evidence of degradation were used for downstream analysis. Total RNA was standardized to uniform concentration and reverse transcribed into complementary DNA (cDNA) using a high-capacity cDNA synthesis kit according to the manufacturer’s instructions.

Quantitative real-time PCR (qPCR) was performed using SYBR Green Master Mix (Applied Biosystems™, Waltham, MA, USA) on a CFX Opus 384 Real-Time qPCR System (Bio-Rad Laboratories, Hercules, CA, USA). Each sample was analyzed in triplicate. The thermal cycling conditions consisted of an initial denaturation at 95 °C for 10 min, followed by 40 cycles of denaturation at 95 °C for 15 s, gene-specific annealing for 20 s, and extension at 72 °C for 15 s, as previously described [25]. A melting curve analysis was conducted to confirm amplification specificity. Relative gene expression was calculated using the 2^−ΔΔCt^ method [26], with normalization to the geometric mean of two housekeeping genes, GAPDH and β-actin (ACTB), to obtain ΔCt values. For each target gene, the sample with the lowest relative expression was used as the calibrator, such that ΔΔCt = ΔCt(sample) − ΔCt(calibrator). Accordingly, the calibrator sample has a relative expression value of 1, and all other samples are expressed relative to it. The control group was not used as the calibrator. Primer sequences for the target and reference genes are presented in Table 1. A no-template control (NTC) was included in each run to verify the absence of contamination.

### 2.6. Statistical Analysis

Histomorphological measurements and relative gene expression data were analyzed using SAS software (version 9.24; SAS Institute Inc., Cary, NC, USA). The replicate cage served as the experimental unit for all analyses. Two birds were sampled from each replicate for histological and gene expression measurements, and the average value of the two birds was used to represent that replicate in the statistical analysis. Data was analyzed using one-way analysis of variance (ANOVA) under a completely randomized design according to the model:

*Y_ij_* = *μ* + *T_i_* + *e_ij_*

where *Y_ij_* represents the observed response variable, *μ* is the overall mean, *T_i_* is the fixed effect of dietary treatment, and *e_ij_* is the residual error. Prior to analysis, the assumptions of ANOVA were verified: the normality of model residuals was assessed using the Shapiro–Wilk test and visual inspection of normal quantile–quantile (Q-Q) plots, and homogeneity of variance among treatments was evaluated using Levene’s test.

When a significant treatment effect was detected, means were separated using Tukey’s multiple comparison test. Orthogonal polynomial contrasts were used to evaluate linear and quadratic responses to increasing dietary BMS inclusion levels. Because BMS was included at graded levels (0, 2, 4, and 6%), these two approaches were applied to the same data to address different questions. Tukey’s test identified which specific treatment means differed from one another, whereas the polynomial contrasts characterized the form of the dose–response relationship across increasing inclusion levels. Both analyses are therefore reported together. Data are presented as mean ± standard error (SE). Statistical significance was declared at *p* < 0.05.

For the integrative evaluation of barrier-related gene expression, the two birds sampled per cage were first averaged so that the replicate cage served as the experimental unit (n = 5 per treatment). Relative gene expression values (2^−ΔΔCt^) were then standardized gene-wise to z-scores across the cage-level values. These composite indices were not adopted from previously validated scoring systems but were defined as exploratory integrative summaries of barrier-related gene expression, newly developed for the present study to provide a single integrated view of the coordinated transcriptional response across multiple barrier-related genes, intended to supplement, rather than replace, the gene-level analyses. Two composite indices were calculated: (i) a Tight Junction Index (TJI), the mean of the *TJP1*, *TJP2*, *OCLN*, and *CLDN1* z-scores, and (ii) a Barrier Index, the mean of those four plus *MUC2*. All constituent genes are positively oriented with respect to barrier integrity; accordingly, all z-scores shared the same directional convention, no sign inversion was required, and higher index values correspond to a more favorable barrier-related expression profile. All markers were assigned equal weight, as there is no established a priori basis for differential weighting. Because index data were not normally distributed, treatment differences were evaluated using the Kruskal–Wallis test, followed where applicable by Dunn’s post hoc test with Benjamini–Hochberg correction (two-sided, α = 0.05). Analyses and visualization were conducted in R (version 4.1.2). No observations were missing, so no data was excluded from either index.

## 3. Results

### 3.1. Effects of Brown Mushroom Stem (BMS) Supplementation on Ileum Histomorphology

**Figure 3:**
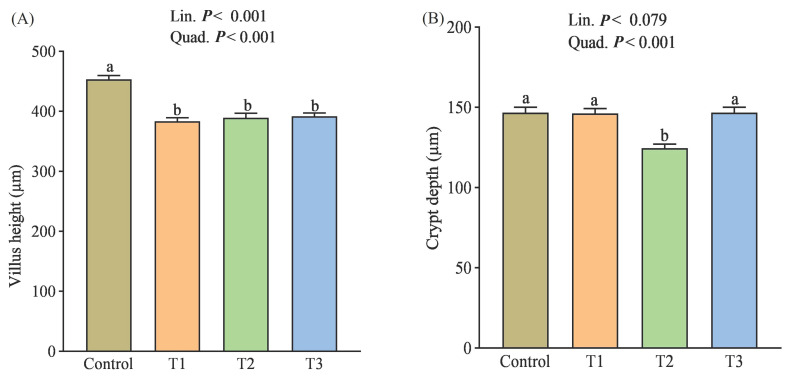
Effects of dietary brown mushroom stem (BMS) supplementation on ileal histomorphology in layer chicks. (**A**) Villus height (VH) and (**B**) crypt depth (CD). Treatments were Control (0% BMS), T1 (2% BMS), T2 (4% BMS), and T3 (6% BMS). Data are presented as the mean ± standard error of the mean (SEM); n = 5 replicate cages per treatment. Means were compared using Tukey’s multiple comparison test; different superscript letters (a, b) above bars denote significant differences among treatments (*p* < 0.05), whereas bars sharing the same letter do not differ significantly. Linear (Lin.) and quadratic (Quad.) polynomial contrasts were used to evaluate the dose–response to increasing BMS inclusion levels, and the corresponding *p*-values are shown above each panel.

The effects of dietary BMS supplementation on small intestinal histomorphology are presented in Figure 3. Villus height (VH; Figure 3A) was significantly influenced by dietary treatment, with all BMS-supplemented groups exhibiting significantly lower VH compared with the control group (linear and quadratic effects, *p* < 0.001). No significant differences in VH were observed among the BMS-supplemented groups. Crypt depth (CD; Figure 3B) was altered by dietary treatment, showing a significant quadratic response (*p* < 0.001), whereas the linear contrast was not significant (*p* = 0.079). The T2 group exhibited a significantly lower CD than the control, T1, and T3 groups, whereas no significant differences were observed among the control, T1, and T3 groups.

### 3.2. Effects of Brown Mushroom Stem (BMS) Supplementation on Tight Junction Gene Expression

**Figure 4:**
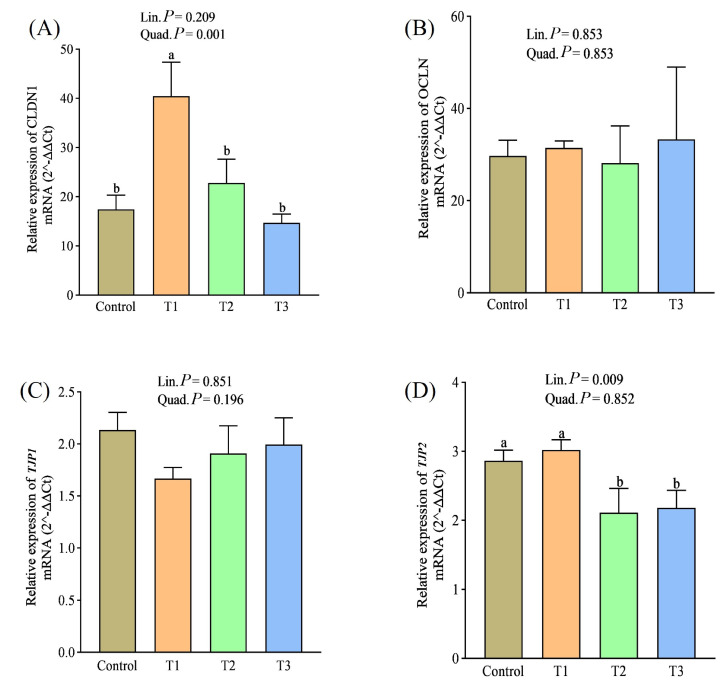
Effects of dietary brown mushroom stem (BMS) supplementation on the relative mRNA expression of tight junction-related genes in the ileum of layer chicks. (**A**) *CLDN1*, (**B**) *OCLN*, (**C**) *TJP1*, and (**D**) *TJP2*. Treatments were Control (0% BMS), T1 (2% BMS), T2 (4% BMS), and T3 (6% BMS). Relative gene expression was calculated using the 2^−ΔΔCt^ method and normalized to the geometric mean of *GAPDH* and *ACTB* (β-actin). Data are presented as the mean ± standard error of the mean (SEM); n = 5 replicate cages per treatment. Means were compared using Tukey’s multiple comparison test; different superscript letters (a, b) above bars denote significant differences among treatments (*p* < 0.05), whereas bars sharing the same letter, or panels without letters, did not differ significantly. Linear (Lin.) and quadratic (Quad.) polynomial contrasts were used to evaluate the dose–response to increasing BMS inclusion levels, and the corresponding *p*-values are shown above each panel.

The effects of dietary BMS supplementation on the relative mRNA expression of tight junction-associated genes in the small intestine are shown in Figure 4. The expression of *CLDN1* mRNA (Figure 4A) was significantly affected by dietary treatment, exhibiting a significant quadratic response (*p* = 0.001), whereas the linear effect was not significant (*p* = 0.209). The T1 group showed significantly higher *CLDN1* mRNA expression compared with the control, T2, and T3 groups. The expression of *OCLN* mRNA (Figure 4B) was not influenced by dietary treatment, as neither linear nor quadratic contrasts were significant (*p* = 0.853 for both). Similarly, the expression of *TJP1* mRNA (Figure 4C) was not affected by BMS supplementation, with no significant linear or quadratic responses observed (*p* = 0.851 and *p* = 0.196, respectively). In contrast, the expression of *TJP2* mRNA (Figure 4D) was significantly influenced by dietary treatment, showing a significant linear effect (*p* = 0.009) but no quadratic response (*p* = 0.852). The control and T1 groups exhibited significantly higher *TJP2* mRNA expression compared with the T2 and T3 groups.

### 3.3. Effects of Brown Mushroom Stem (BMS) Supplementation on Goblet Cells and MUC2 mRNA Expression

**Figure 5:**
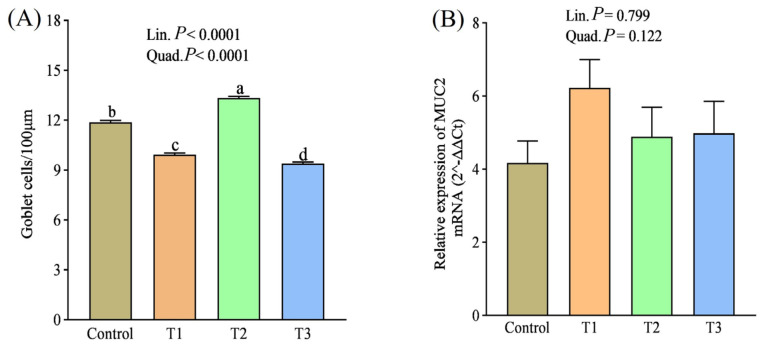
Effects of dietary brown mushroom stem (BMS) supplementation on (**A**) goblet cell density and (**B**) relative mRNA expression of *MUC2* in the ileum of layer chicks. Treatments were Control (0% BMS), T1 (2% BMS), T2 (4% BMS), and T3 (6% BMS). Goblet cell density is expressed as the number of cells per 100 µm of villus epithelial surface. Relative gene expression was calculated using the 2^^−ΔΔCt^ method and normalized to the geometric mean of *GAPDH* and *ACTB* (β-actin). Data are presented as the mean ± standard error of the mean (SEM); n = 5 replicate cages per treatment. Means were compared using Tukey’s multiple comparison test; different superscript letters (a, b, c, d) above bars denote significant differences among treatments (*p* < 0.05), whereas panels without letters showed no significant differences. Linear (Lin.) and quadratic (Quad.) polynomial contrasts were used to evaluate the dose–response to increasing BMS inclusion levels, and the corresponding *p*-values are shown above each panel.

Dietary BMS supplementation significantly affected goblet cell density in the ileum (Figure 5A), with both linear and quadratic responses being significant. Goblet cell density in T2 was significantly higher than in the Control, T1 and T3 groups. The Control group showed significantly greater goblet cell density than both T1 and T3, and T1 was significantly higher than T3. The relative mRNA expression of *MUC2* in the ileum is presented in Figure 5B. Dietary treatment did not significantly influence *MUC2* mRNA expression, as neither linear nor quadratic contrasts were significant.

### 3.4. Effects of Brown Mushroom Stem (BMS) Supplementation on the Intestinal Barrier Indices

**Figure 6:**
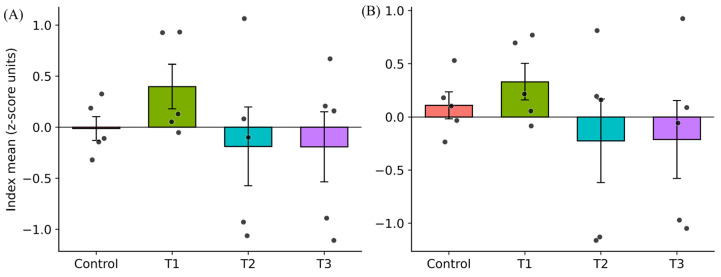
Integrated intestinal barrier indices in layer chicks fed diets containing brown mushroom stem (BMS). (**A**) Barrier Index (combined standardized expression of *CLDN1*, *OCLN*, *TJP1*, *TJP2*, and *MUC2*) and (**B**) Tight Junction Index (the four tight junction genes only). Treatments included Control (0% BMS), T1 (2% BMS), T2 (4% BMS), and T3 (6% BMS). Bars represent the group mean and error bars the standard error (SEM); black points show the individual cage values (n = 5 replicate cages per treatment), with the replicate cage as the experimental unit. Indices are expressed in z-score units. Neither index differed significantly among treatments (Kruskal–Wallis, *p* > 0.05).

The Barrier Index and the Tight Junction Index (TJI) are presented in Figure 6. Neither index differed significantly among treatments (Barrier Index: H = 1.94, df = 3, *p* = 0.586; Tight Junction Index: H = 1.42, df = 3, *p* = 0.700; Kruskal–Wallis), and no pairwise comparison was significant after Benjamini–Hochberg correction. The T1 group showed numerically higher index values than the other groups, consistent with the individual gene-level results.

## 4. Discussion

This study was designed to test the hypothesis that brown mushroom stem (BMS), a fiber- and bioactive-rich soybean meal replacer, modulates ileal barrier-related gene expression in a dose-dependent manner, coordinating epithelial structural remodeling with transcriptional regulation of tight junction and mucin-related genes. The present findings demonstrate that BMS does not act as a simple structural modifier but rather induces regulated epithelial adaptation. The ileum responded differently across inclusion levels, indicating a biologically controlled recalibration of morphology, proliferative activity, and barrier-associated gene expression.

The reduction in villus height observed across all BMS-supplemented groups indicates that the ileal mucosa underwent consistent architectural remodeling in response to dietary inclusion. The intestinal epithelium is one of the most dynamic tissues in poultry, continuously exposed to luminal stimuli and rapidly adapting to dietary composition [27,28]. Villus shortening is commonly reported when birds consume diets enriched with insoluble fiber or altered nutrient density [29]. Because villus height reflects absorptive surface area, the consistent reduction across all BMS-supplemented groups may indicate a decrease in absorptive capacity, and not structural recalibration alone. Villus height is also influenced by luminal viscosity, mechanical stimulation, nutrient availability, and microbial fermentation dynamics, and a decrease may partly reflect adjustment to the increased non-digestible fraction of the diet [30]. However, because nutrient digestibility and absorption were not measured in the present study, these possibilities cannot be distinguished, and the functional consequence of the reduced villus height remains undetermined. Villus shortening in the present study was not accompanied by crypt hyperplasia. The crypt region represents the proliferative compartment responsible for generating new epithelial cells [16,24,31]. Under conditions of epithelial injury or inflammatory stress, crypt depth typically increases due to accelerated regenerative demand. In contrast, crypt depth exhibited a quadratic response and was the lowest in the 4% group. Reduced crypt depth suggests lower epithelial turnover and decreased proliferative pressure. This pattern indicates that BMS inclusion did not provoke a stress-induced regenerative response or overt epithelial injury at any inclusion level.

Previous poultry studies involving mushroom-derived products have reported mixed histomorphological outcomes. Some reports described increased crypt depth, possibly linked to antinutritional factors such as tannins, phytic acid, or oxalates present in certain mushroom tissues [32,33,34]. Increased crypt depth reflects elevated energy expenditure for tissue renewal and may reduce nutrient utilization efficiency [35,36]. The absence of crypt deepening in the present study suggests that BMS did not impose substantial epithelial irritation or toxic stress. Instead, the structural changes likely reflect controlled adaptation to dietary fiber and mushroom-derived components rather than pathological injury.

While morphology provides insight into epithelial architecture and turnover, it does not directly define functional barrier integrity. Tight junction (TJ) proteins regulate paracellular permeability and form the primary defense against microbial translocation [21,37]. Increased intestinal permeability has been associated with immune activation, coccidial susceptibility, bacterial movement across the epithelium, and reduced performance in poultry [38,39]. Therefore, evaluating TJ gene expression is essential to determine whether structural remodeling compromises or strengthens epithelial sealing. Among the TJ genes analyzed, *CLDN1* mRNA expression demonstrated a significant quadratic response, with the highest expression observed in the 2% group. Claudin-1 is a sealing claudin that contributes to tight junction strand formation and restricts paracellular ion movement [40]. At the protein level, claudin-1 contributes to epithelial tightness and reduced paracellular permeability. Furthermore, *CLDN1* mRNA upregulation has been associated with epithelial repair and barrier reinforcement processes [41]. The higher *CLDN1* mRNA expression at moderate BMS inclusion reflects upregulation of a sealing-associated gene at the transcriptional level; whether this corresponds to enhanced paracellular sealing would need confirmation at the protein and functional levels. This change occurred without concurrent alteration of the other tight junction genes examined, indicating that the transcriptional response was gene-specific rather than global. Importantly, these analyses were limited to mRNA; changes in transcript abundance do not necessarily reflect tight junction protein abundance, localization, or functional barrier integrity, none of which were assessed in the present study and which warrant confirmation in future work.

*OCLN* and *TJP1* mRNA expression remained stable across treatments. Occludin functions as a regulatory transmembrane component that modulates paracellular permeability, whereas *ZO-1* anchors transmembrane proteins to the actin cytoskeleton and maintains structural cohesion [37]. Stability of these genes is mechanistically important because disruption of TJ architecture typically manifests as downregulation or dysregulation of these components. Their unchanged expression indicates that the transcription of these barrier-related genes was maintained across all BMS inclusion levels.

The linear decline in *TJP2* mRNA expression with increasing BMS inclusion introduces an additional layer of interpretation. *ZO* proteins serve as cytoplasmic scaffolding adaptors linking claudins and occludin to the actin cytoskeleton [42]. Alterations in *TJP2* mRNA may reflect cytoskeletal remodeling rather than permeability loss. Under increased fiber load, luminal mechanical forces and changes in epithelial tension may induce reorganization of cytoskeletal interactions. Such remodeling could reduce *TJP2* transcription without disrupting overall tight junction sealing, particularly when *CLDN1* mRNA expression remains elevated at lower inclusion levels. Thus, the observed *TJP2* mRNA expression decreases likely represents structural fine-tuning rather than barrier failure.

Goblet cell dynamics provide further mechanistic insight. Goblet cells synthesize and secrete mucins that form the mucus layer, which act as a physical and biochemical shield protecting tight junctions [43]. Goblet cell differentiation and mucus secretion are influenced by luminal microbial signals and dietary components [44]. The quadratic increase in goblet cell density in the 4% group suggests that intermediate BMS inclusion stimulated secretory lineage expansion. This may represent an adaptive response to increased fiber content, preparing the mucosa for enhanced lubrication and protection. However, *MUC2* mRNA expression remained statistically unchanged across treatments. *MUC2* is the principal gel-forming mucin in the intestine, and reduced expression has been associated with mucosal degeneration and spontaneous inflammation [45]. The maintenance of MUC2 transcription across all inclusion levels indicates that mucin gene expression was not suppressed by BMS. The dissociation between goblet cell abundance and *MUC2* mRNA suggests regulation beyond transcriptional control. Goblet cell numbers can increase without immediate upregulation of mucin gene transcription, as mucin production is regulated at translational, storage, and secretion levels. Therefore, the higher goblet cell density at 4% likely reflects expansion of the secretory cell population rather than increased mucin gene transcription. As this increase was not accompanied by higher *MUC2* mRNA, it represents a change in goblet cell number rather than demonstrable enhancement of mucus barrier function.

The integrated intestinal barrier indices further indicate that BMS supplementation did not cause a coordinated change in intestinal barrier-related gene expression. Tight junction proteins are key regulators of epithelial barrier integrity and paracellular permeability [19,21,37], while *MUC2* is a major mucus barrier component produced by goblet cells [44,45]. In the present study, *CLDN1* mRNA expression increased in the 2% BMS group, whereas *TJP2* mRNA expression decreased at higher inclusion levels. However, *OCLN*, *TJP1*, and *MUC2* mRNA expressions remained unchanged. Consequently, neither the Barrier Index nor the Tight Junction Index differed significantly among treatment groups. This suggests that the transcriptional response to BMS was marker-specific rather than a uniform change across the selected barrier gene set. These indices, however, reflect only barrier-related gene expression and not functional barrier integrity, which was not assessed. The unchanged indices therefore indicate the absence of a coordinated transcriptional shift rather than confirmed preservation of barrier function. At 2% inclusion, the higher *CLDN1* mRNA expression represents a limited, marker-specific transcriptional response, whereas higher BMS levels did not produce additional changes in barrier-related gene expression.

When structural and molecular data are considered collectively, a consistent dose-dependent pattern emerges. At low inclusion, BMS enhanced *CLDN1* mRNA expression, a transcriptional upregulation of a sealing-associated gene, while structural stability was maintained. At intermediate inclusion, epithelial proliferation was minimized, and goblet cell density was the highest, indicating secretory and structural adaptation without further changes in tight junction gene expression. At high inclusion, villus shortening persisted, and *TJP2* mRNA expression declined, yet no additional improvement in sealing gene expression occurred. These patterns indicate that BMS acts as a dose-dependent modulator of ileal morphology and barrier-related gene expression, with a threshold beyond which additional inclusion is associated with structural adaptation rather than further transcriptional change. As paracellular permeability and tight junction protein abundance were not measured, these interpretations are restricted to the morphological and transcriptional levels.

However, the intestine must balance absorptive surface area, barrier tightness, and metabolic cost. Increased fiber may reduce effective nutrient density, prompting structural economization, while bioactive compounds may simultaneously enhance barrier signaling. The present findings suggest that moderate inclusion levels optimize this balance by increasing sealing-associated gene expression without imposing excessive structural compromise. Excessive inclusion shifts the intestine toward architectural adjustment without additional changes in barrier-related gene expression. BMS supplementation induced regulated epithelial recalibration rather than pathological alteration. The intestine showed stable tight junction gene expression, maintained *MUC2* transcription, and selectively higher *CLDN1* expression at moderate inclusion levels. These findings emphasize the importance of defining inclusion thresholds when incorporating mushroom-derived by-products into poultry diets. Appropriate dosing allows exploitation of sustainability benefits while preserving epithelial structure and barrier-related gene expression.

A limitation of the present study is that it was conducted in healthy, unchallenged layer chicks under standardized, low-stress husbandry conditions. This design was chosen deliberately to characterize the baseline morphological and transcriptional response of the ileum to graded BMS inclusion, without the confounding influence of an immune or enteric challenge, and the findings should therefore be interpreted within this context. Because barrier-related genes are most strongly engaged under conditions of physiological or pathogenic stress, the transcriptional responses observed here may not fully reflect how BMS would influence gut integrity when the barrier is actively challenged. Consequently, the present results establish whether BMS modulates barrier-related gene expression under homeostatic conditions, but they cannot establish whether these changes confer functional protection. Future studies employing challenge models such as enteric pathogen infection (e.g., *Eimeria* or *Salmonella*), heat stress, or dietary stressors combined with protein level and functional barrier assessment are necessary to determine whether the transcriptional changes reported here translate into tangible improvements in gut health and resilience under stress. A further consideration is that the dietary intervention was initiated at 3 weeks of age, after the initial rapid phase of post-hatch intestinal development; the present findings therefore describe the response of the maturing ileum and may not be directly extrapolated to the early post-hatch period, when villus and crypt architecture and barrier maturation are governed largely by intrinsic developmental programming rather than by dietary manipulation.

## 5. Conclusions

Brown mushroom stem (BMS) supplementation exerted dose-dependent effects on ileal structure and barrier-associated gene expression in layer chicks that varied in both magnitude and direction with inclusion level. Although villus height was reduced across BMS-fed groups, crypt depth was not increased in any group and was the lowest at 4% inclusion. Because crypt deepening is a typical sign of epithelial stress, its absence suggests the villus reduction occurred without overt mucosal injury. As nutrient digestibility was not measured, the functional impact of the reduced villus height remains to be determined. Treatment with 2% BMS increased *CLDN1* mRNA expression, a selective, marker-specific change in a sealing-associated gene, while *OCLN*, *TJP1*, and *MUC2* mRNA expression remained unchanged. The integrated barrier index did not differ significantly among treatments, indicating the absence of a coordinated change in barrier-related gene expression. Higher inclusion levels altered ileal morphology without further changes in barrier-related gene expression. Overall, within the scope of ileal morphology and the selected barrier-related mRNA markers examined, BMS can serve as a sustainable partial soybean meal replacer, with the lowest inclusion level (2%) showing the most favorable barrier-related gene expression. The most favorable transcriptional response was thus confined to the lowest inclusion level, with no additional benefit observed at 4% or 6% inclusion. As functional barrier integrity was not directly measured, these conclusions are limited to morphological and transcriptional outcomes.

## Figures and Tables

**Table 1:** Primer sequences used for quantitative real-time PCR.

Genes	Primer	Sequence (5′-3′)	Amplicon Size (bp)	Accession Number
*TJP1*	Forward	GCTGGACAGTTTGAGCCTTC	156	XM_015278975.4
	Reverse	TGGTTTCATGGCTGGATCCT		
*TJP2*	Forward	AGTCCACCTCCAGCATTCAA	165	NM_204918
	Reverse	CACAGAAACAGGTGGTGGTG		
*OCLN*	Forward	ATCGTCTACATCATGGGCGT	180	NM_205128.1
	Reverse	GACGATGAGGAACCCACAGA		
*CLDN1*	Forward	TGTGTTCAGAGGCATCAGGT	196	NM_001013611.2
	Reverse	ACCCAGCTCAAGTACAGGTC		
*MUC2*	Forward	TGTGTTTGAGAAGTGCCGTG	160	XM_040673077.2
	Reverse	ACTTCTCCAGTCAACGCAGA		
*GAPDH*	Forward	CATCCAAGGAGTGAGCCAGG	187	NM_204305.2
	Reverse	TATCAGCCTCTCCCACCTCC		
*ACTB*	Forward	AGTACCCCATTGAACACGGT	197	NM_205518.2
	Reverse	ATACATGGCTGGGGTGTTGA		

*TJP1* = tight junction protein 1; *TJP2* = tight junction 2; *OCLN* = occludin; *CLDN1* = claudin-1; *MUC2* = mucin-2; *GAPDH* = glyceraldehyde-3-phosphate dehydrogenase; *ACTB* = β-actin. *GAPDH* and *ACTB* were used as reference genes for normalization.

## Data Availability

The original contributions presented in this study are included in the article. Further inquiries can be directed to the corresponding author.

## Appendix A

**Table A1:** Ingredient and calculated chemical composition of the experimental diets containing graded levels of brown mushroom stem (BMS) as a partial replacement for soybean meal (as-fed basis).

Item	Control (0% BMS)	2% BMS	4% BMS	6% BMS
***Ingredients (%)***				
Corn	66.000	66.000	66.000	66.000
Soybean meal, 48%	25.887	25.369	24.852	24.334
Brown mushroom stem (19.63% CP)	0.000	0.518	1.035	1.553
Soybean oil	1.000	1.000	1.000	1.000
Vitamin premix ^a^	0.050	0.050	0.050	0.050
Trace mineral premix ^a^	0.100	0.100	0.100	0.100
Limestone	2.200	2.200	2.200	2.200
Monocalcium phosphate	1.733	1.733	1.733	1.733
Salt	2.700	2.700	2.700	2.700
Sodium bicarbonate	0.133	0.133	0.133	0.133
DL-methionine	0.197	0.197	0.197	0.197
Total	100.000	100.000	100.000	100.000
***Calculated chemical composition***				
Metabolizable energy (MJ/kg)	11.798	11.782	11.767	11.751
Crude protein (%)	18.036	17.889	17.742	17.595
Crude fiber (%)	2.358	2.389	2.414	2.442
Crude fat (%)	3.767	3.767	3.768	3.768
Calcium (%)	1.199	1.098	1.097	1.095
Phosphorus (%)	0.746	0.743	0.742	0.737
Lysine (%)	0.953	0.941	0.929	0.917
Methionine (%)	0.466	0.463	0.460	0.456

BMS, brown mushroom stem; CP, crude protein. Diets were formulated to be isonitrogenous and isocaloric and to meet or exceed the nutrient requirements for layer chicks (NRC, 1994 [46]). ^a^ The vitamin–mineral premix was supplemented at the following levels per kilogram of diet: 8,818,342 IU vitamin A; 3,086,420 IU vitamin D3; 36,742 IU vitamin E; 13 mg vitamin B12; 1177 mg menadione; 4775 mg riboflavin; 16,168 mg D-pantothenic acid; 2350 mg thiamine; 36,742 mg niacin; 5732 mg vitamin B6; 1398 mg folic acid; 104,460 mg choline; 441 mg biotin; 120 mg manganese (Mn); 1.4 mg copper (Cu); 120 mg zinc (Zn); 120 mg iron (Fe); 0.5 mg selenium (Se); and 800 mg iodine (I).
